# Energy Transition Pathways for Deep Decarbonization of the Greater Montreal Region: An Energy Optimization Framework

**DOI:** 10.3390/en15103760

**Published:** 2022-05-20

**Authors:** Sajad Aliakbari Sani, Azadeh Maroufmashat, Frédéric Babonneau, Olivier Bahn, Erick Delage, Alain Haurie, Normand Mousseau, Kathleen Vaillancourt

**Affiliations:** 1 GERAD (Group for Research in Decision Analysis) and Department of Decision Sciences, HEC Montréal, Montréal, QC H3T 2A7, Canada; sajad.aliakbarisani@hec.ca (S.A.S.); azadeh.maroufmashat@hec.ca (A.M.); erick.delage@hec.ca (E.D.); ahaurie@gmail.com (A.H.); 2 Department of Operations Management-Supply Chain-Information Systems, KEDGE Business School, 680 Cr de la Libération, 33405 Talence, France; frederic.babonneau@kedgebs.com; 3 ORDECSYS, 4 Place de l’Etrier, CH-1224 Chêne-Bougeries, Switzerland; 4 Geneva School of Economics and Management, University of Geneva, Boulevard du Pont-d’Arve 40, CH-1211 Geneva, Switzerland; 5 Département de Physique, Faculté des Arts et des Sciences, Université de Montréal, Montréal, QC H3T 1J4, Canada; normand.mousseau@umontreal.ca; 6 ESMIA Consultants, Blainville, QC J7B 6B4, Canada; kathleen@esmia.ca

**Keywords:** bottom–up energy model, cities, deep decarbonization, energy policy, ETEM

## Abstract

More than half of the world’s population live in cities, and by 2050, it is expected that this proportion will reach almost 68%. These densely populated cities consume more than 75% of the world’s primary energy and are responsible for the emission of around 70% of anthropogenic carbon. Providing sustainable energy for the growing demand in cities requires multifaceted planning approach. In this study, we modeled the energy system of the Greater Montreal region to evaluate the impact of different environmental mitigation policies on the energy system of this region over a long-term period (2020–2050). In doing so, we have used the open-source optimization-based model called the Energy–Technology–Environment Model (ETEM). The ETEM is a long-term bottom–up energy model that provides insight into the best options for cities to procure energy, and satisfies useful demands while reducing carbon dioxide (CO_2_) emissions. Results show that, under a deep decarbonization scenario, the transportation, commercial, and residential sectors will contribute to emission reduction by 6.9, 1.6, and 1 million ton CO_2_-eq in 2050, respectively, compared with their 2020 levels. This is mainly achieved by (i) replacing fossil fuel cars with electric-based vehicles in private and public transportation sectors; (ii) replacing fossil fuel furnaces with electric heat pumps to satisfy heating demand in buildings; and (iii) improving the efficiency of buildings by isolating walls and roofs.

## 1. Introduction

Human activities in recent years have increased levels of greenhouse gases (GHG), including carbon dioxide (CO_2_), resulting in global warming. A projection of the current trends [1] shows that the global temperature may exceed the Paris Accord goal of 1.5 °C by 2030. To avoid dangerous disruptions in the climate system, we must implement urgent mitigation measures.

Energy production is among the most important human activities that are responsible for more than three quarters of global GHG emissions [2]. Therefore, the decarbonization of energy systems is one of the most important measures against global warming. On the other hand, cities, as the main consumers of energy commodities, can play an important role in GHG reduction. Currently, over 55% of humans live in urban areas, and it is expected that this proportion will reach 68% by 2050 [3]. The economic activities in cities form nearly 80% of the global gross domestic production (GDP), and they consume more than 75% of the world’s primary energy, and emit more than 70% of anthropogenic carbon [4]. Studies show that a proper city-level energy management can potentially reduce emissions from urban buildings and transportation systems up to 90% by 2050 [5]. Therefore, in recent years, significant actions have been taken by many cities, sub-national states, and private sectors to mitigate emissions at an urban scale. A summary of these actions can be found in [6] for Portuguese municipalities, in [7] for European municipalities, and in the “Climate Innovation Program” [8] for Canada.

This paper proposes a framework to model and analyze the energy transitions pathways of the Greater Montreal (GM) region in the province of Quebec in Canada. The province of Quebec plans to reduce its GHG emissions to 37.5% of their 1990 level by 2030, and to reach carbon neutrality by 2050 [9]. To this end, Quebec has developed a green economy plan for 2020–2030, with the purpose of stimulating the economy, creating jobs, etc., while reducing GHG emissions. Specifically, the government of Quebec plans to (i) spend over 6 billion dollars during the first five years (i.e., 2021–2026) to accelerate the widespread deployment of electrification for infrastructures in transportation, industrial, residential, and commercial sectors; (ii) promote the utilization of bioenergies, renewables, natural gases, and hydrogen for decarbonizing the heating demand in commercial and industrial sectors; and (iii) improve the performance of final energy consumption technologies and energy efficiency [10]. However, these provincial plans need to be broken down into city-level targets in order to become achievable. The purpose of the current study is to analyze the entire energy system of the GM region to highlight the potentials of GHG reductions by considering different technological options on both the supply and demand sides.

Our methodology is to optimize the configuration of energy systems at an urban level. Specifically, we adapt the formulation of the open-source Energy–Technology–Environment Model (ETEM) [11] to model the capacity expansion of generation technologies, as well as the final energy consumption technologies in different sectors. The ETEM minimizes the total energy system costs, including investment, fixed, and variable operational costs, while satisfying demand and environmental constraints. One salient feature of the ETEM is its ability to consider necessary details on the demand side, such as how energy is used by consumers, and how electrical networks perform demand response programs. Such a detailed emphasis on the demand-side technologies makes this model well-suited to analyze the energy value chains and different GHG-reduction policies at a city level. Finally, we perform a sensitivity analysis to evaluate the impact of different GHG-reduction scenarios on the expansion of the energy sector in the GM region. The results and analysis in this paper can be used by policy makers in the Greater Montreal region, but also in other cities where electricity is mostly from renewable sources of energy. In addition, the methodology presented in this paper (developing a reference energy system and optimizing the resulting capacity expansion problem based on the ETEM approach) can be followed to analyze the energy system of any other city.

The remainder of this paper is structured as follows. Section 2 provides a literature review on related studies. In Section 3, we present the methodology and a description of ETEM. Section 4 elaborates on the definition of scenarios. Section 5 describes the results, and Section 6 provides further discussion on our major findings. Finally, we conclude in Section 7.

## 2. Literature Review

In this section, we first review recent publications that study the impacts of environmental policies on energy systems. Specifically, we investigate their methods and their case studies. Then, we give a summary of the history of the ETEM, and introduce the most important case studies that have been analyzed using the ETEM.

One related stream of research is the study of energy transition and the evaluation of the environmental impact of a single sector, such as transportation [12], buildings [13], or power distribution networks [14]. Forsberg et al. [15] developed a local energy transition model for transportation sector using the TIMES-City model in Sweden. Yuan et al. [16] investigated technoeconomic impacts of the electrification of transportation systems using energy PLAN for a region in China. Although these studies consider a detailed dynamic of a specific sector, they abstract the interactions between different energy sectors, so they lack a holistic view point. On the other hand, one line of research studies the integration of different sectors, such as electricity and heating, to support the energy transition in cities [17]. Another study also investigates the optimal design of urban multi-energy hub networks, including their location, capacity, and other technical characteristics [18], with the purpose of reducing GHG emissions. Although these papers consider the interaction of different energy hubs [19], they ignore the dynamic of investment, and therefore do not provide insight on the expansion strategies in a long-term horizon planning. Another stream of research is to study the energy transition at a national level. Vaillancourt et al. [20] investigate decarbonization pathways for Canada until 2050 using the “North American TIMES Energy Model” (NATEM). Shakouri et al. [21] develop a framework to consider sustainable development criteria in modeling the energy system at a national level. These national models provide a detailed modeling of the supply technologies; however, in avoiding the curse of dimensionality, they fail to model the demand-side characteristics, such as demand response, detailed description of final energy technologies, and distribution-level electrical network services (e.g., voltage control or reactive power compensations).

Energy planning of an urban scale requires a detailed modeling of how the final energy is used in cities, what the possible options are to satisfy energy services, and how the consumption behavior in one section might affect the energy consumption in other sections. To this end, urban energy planning models have a holistic viewpoint that integrates different sectors, such as transportation, residential, commercial, etc. In [22,23], a review of methods and approaches to model the energy system at an urban scale is presented. Dagoumas [24] explores the interconnection between socioeconomics, energy, and environmental components for London. Xydis [25] develop an optimization model to identify the optimal strategy to meet the energy requirements of Athens while satisfying technoeconomic constraints. Elizondo et al. [26] evaluates different decarbonization scenarios of the energy system in Mexico City by 2050 using an integrated approach. Finally, Lind and Espegren [27] use the TIMES model to design an optimal low-carbon pathway for the energy system of the city of Oslo, Norway. However, it remains that there is only a limited number of papers carrying out research at the city level. This paper contributes to this limited body of literature by proposing an application for the city of Montreal.

In this paper, we use the ETEM to analyze the energy system of the GM region and evaluate different energy transition pathways. The ETEM provides the planner with a decision-making framework that not only considers a detailed description of the useful demand technologies, but also models some important characteristics of the demand side, such as demand response in electrical networks. The ETEM was first formulated in [28,29] to model the energy system of the Midi-Pyrénées region in France and the Arc Lémanique region in Switzerland. In [11,30], the ETEM model was further developed by introducing a linear approximation of power flow constraints and distribution module. In addition, the new formulation, called the ETEM-SG, explicitly modeled the reserve capacities provided by demand response and ancillary services, such as reactive power compensation offered by flexible loads. In [11,31], a version of the ETEM-SG is presented that considers the uncertainty of investment costs and of availability of technologies and transmission capacity lines. A static robust optimization approach is used to solve the problem. In [32], a robust multiperiod formulation of the ETEM, with uncertain demand response, is developed to better control the level of conservatism in planning. Finally, in [33,34], two robust versions of the ETEM are linked to a mean field game model that presents the charging behavior of a large fleet of electric vehicles (EVs). This paper is an original application of the ETEM for the energy database of the GM region.

## 3. Methodology

This research paper proposes a model for optimal expansion planning of the existing and future technologies in the energy sector of the GM region up to 2050, while meeting GHG emission reduction targets. To do so, we use the formulation of the Energy–Technology–Environment Model (ETEM). Introduced for the first time in [28,29], the ETEM is an open-source energy model designed to analyze energy systems at an urban scale. For example, the ETEM has been applied to model the energy system of the Midi-Pyrénées region in France [28] and the Arc Lémanique region in Switzerland [11]. We slightly modified the formulation of the ETEM to adapt it to the Montreal database. For example, contrary to the original version which calculates GHG emissions at the process level, we calculated emissions based on the total primary energy consumption in the entire system.

### 3.1. ETEM Model Description

The ETEM is an open-source, long-term, regional, bottom–up energy model cast as a linear programming problem. The ETEM belongs to the MARKAL-TIMES family of models, and in a more general framework it is a capacity expansion problem. The ETEM models the entire energy sector from primary resources to useful demands and suggests the best combination of generation and end-user technologies to satisfy the growing demands at a minimum cost.

In the ETEM, the planning horizon is typically 30–50 years which includes several decision-making periods. Each decision-making period typically represents a duration of 5 years. Moreover, in order to capture the consumption and generation short-term patterns, each year is divided into several time slices with similar demand loads. For example, Babonneau et al. [31] divide each year into 3 seasons, and a typical day in each season is divided into 4 load-parts: morning, peak-day, peak-night, and night; therefore, in total, they introduce 12 time slices in a year that capture different demand load patterns. However, in this research we are modeling higher-resolution time slices with hourly load parts. In other words, as the useful demands and energy production are influenced by different weather conditions in different seasons (e.g., demand for heating in residential sector is higher in winter) and time of the day (e.g., during peak hours, the demand for electricity is higher than during other parts of the day), we have considered four seasons, including winter, spring, summer and autumn. In addition, each season is represented with a typical day with 24 hourly load parts. Therefore, we consider in total 96 time slices in a year.

The objective function in the ETEM is to minimize the total discounted cost of the system over the entire planning horizon. The total cost consists of (i) investment cost, (ii) fixed and variable operational and maintenance cost, (iii) net import and export cost, (iv) energy transmission cost, and finally (v) the salvage value that considers the end-of-life value of the retired technologies. The model provides insights on the optimal capacity expansion and the optimal level of production for each technology as well as the optimal level of import and export for each primary energy or energy resource. In addition, the ETEM gives insights on the optimal level of demand response by flexible loads. The demand response, which is defined as shifting the electricity demand from peak to off-peak time slices, reduces the need to build extra reserved capacity in the system.

Different constraints in the ETEM are categorized into technical, network, environmental, and economic constraints. Technical constraints guarantee that the solution of the model meet technical characteristics of the generation technologies. For example, technology efficiency and capacity factor constraints limit the level of input and output energy of each technology according to its efficiency and capacity factor. Network constraints guarantee the balance of energy in the entire energy sector. In addition, network constraints limit the energy flow between regions to the capacity of the transmission lines. Environmental constraints limit the total and annual GHG emissions of the system to a desired level. Furthermore, finally, economic constraints refer to a group of constraints that force the solutions of the model to meet realistic economic limitations. For example, market penetration, annual, or total constraints on capacity addition, and annual or total constraint on import or export of the energy commodities, are among the economic constraints that are considered. More details on the ETEM are provided in Appendix A. We also refer interested readers to [28,29] for a complete model description.

### 3.2. Adapting the ETEM to the Greater Montreal Region

We have modeled the energy system of the GM region in the province of Quebec in Canada. This region consists of 5 main subregions, including Montreal agglomeration, Laval, Longueuil, Couronne Nord, and Couronne Sud. The planning horizon runs from 2020 to 2050 and is divided into 6 decision-making periods with a length of 5 years. Moreover, each year has 96 time slices. We used 2015 as a base year to calibrate the model. In 2015, the total energy consumption of the GM region was 642 PJ with 20.8 Mt CO_2_-eq emissions [35]. The region imported 201 PJ of electricity, and generated 34 PJ internally. In the transportation sector, the region consumed 101 PJ, 2.7 PJ, and 0.3 PJ of gasoline, diesel, and electricity, respectively. In total, the transportation sector emitted 6.9 Mt CO_2_-eq. In the residential and commercial sectors, there were 67 and 51 PJ of energy consumed, respectively, resulting in emissions of 2.9 Mt CO_2_-eq.

**Figure 1:**
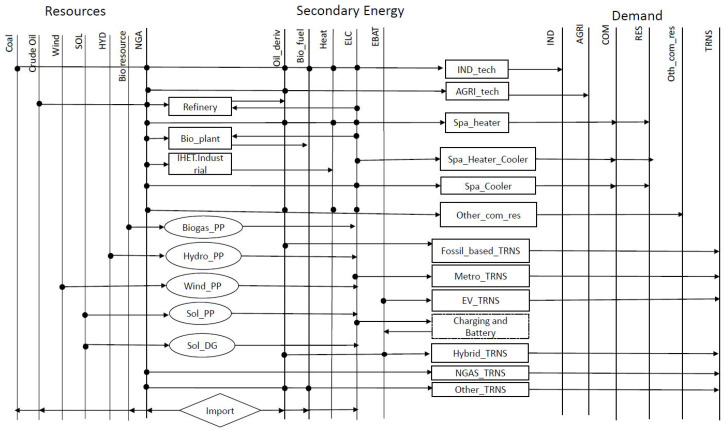
Overview of the reference energy system (RES) in the Greater Montreal region. SOL—solar; HYD—hydro; NGA—natural gas; PP—power plant; DG—distributed generation; ELC—electricity; EBAT—the electricity of batteries; IND—industry; AGRI—agriculture; Spa—space; TRNS—transportation; EV—electric vehicles; RES—residential; COM—commercial.

Figure 1 gives an overview of the reference energy system (RES) in the GM region. In this figure, each horizontal line represents a category of energy commodities, and each box represents a family of conversion technologies. In total, the model includes 51 energy commodities, and 130 conversion technologies in different sectors. We have modeled 13 types of useful demands which are categorized into industrial (IND), agricultural (AGR), commercial (COM), residential (RES), and transportation (TRNS). The useful demand in the transportation sector was divided into 5 modes, including (i) light-duty vehicles, (ii) public transportation, (iii) trains, (iv) metros, and (v) other kinds of transportation (including maritime and air transportation); meanwhile, a generic technology represents the fuel consumption in “other transportation” mode, and a detailed breakdown of all current and possible future technologies were considered for light-duty vehicles, public transportation trains, and metros. Gasoline, natural gas, diesel, and electricity are the main fuels consumed in the transportation sector. Residential and commercial demands include useful energy demand for space heating, space cooling, and “other consumption” (including lighting, electrical appliances, etc.). Similar to transportation, a generic technology represented the energy consumption in the “other consumption” category, but detailed technology choices were modeled for space heating and cooling, including furnaces, different types of heaters, as well as heat pumps. Industrial demand includes coal, natural gas, electricity, heat, diesel, light and heavy fuel oil, propane, and biofuels. Finally, the agricultural sector consumes natural gas, electricity, gasoline, diesel, light and heavy fuel oil, and propane.

Energy commodities are either generated by the already available generation technologies inside the GM region, or imported from the outside. Specifically, we modeled two existing hydropower stations (Beauharnois in Couronne Sud and the Prairies river station in Laval), two existing biogas electricity and heat generation units (one in Couronne Nord and one in the Montreal agglomeration), and the potential to install wind and solar power plants in the north and south of Montreal Island. In addition, we have modeled the Montreal refinery that produces diesel, gasoline, light and heavy fuel oil, propane, and other oil products. Finally, there are two existing bioethanol and biodiesel generation units in Couronne Sud. These generation units can satisfy more than the internal demand and exports part of its production outside the GM region. Beside the internal production, the model also considers the imports of resources and secondary energies. The imported resources include, crude oil, natural gas, and coal. Furthermore, the imported secondary energies include oil products (gasoline, diesel, light and heavy fuel oil, propane, petroleum coke, and jet fuel), ethanol, bioresources (biodiesel, biogas), and electricity.

**Figure 2:**
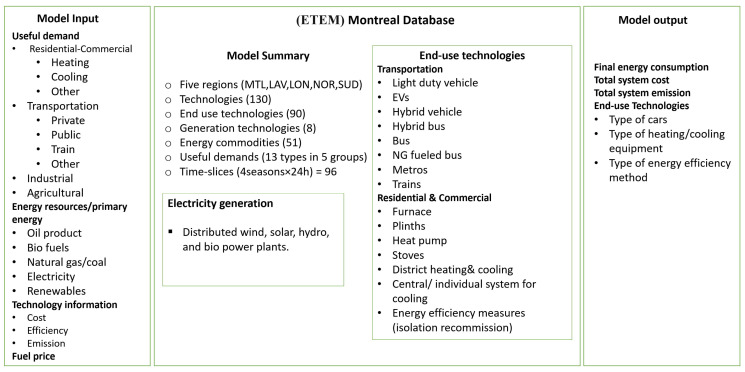
A summary of inputs, outputs, and the main characteristics of the ETEM adapted for the Greater Montreal region.

Figure 2 provides a summary of the important input and output of the ETEM model calibrated for the GM region. The input data includes energy demand, energy prices, resource availability, technical and economical information for technologies, and the carbon content of different fuels. Outputs correspond to the optimal capacity expansion plan, optimal imports and exports of the energy commodities, and the marginal costs of CO_2_ emissions. Further details on the energy database of the GM region are provided in Appendix B.

As previously indicated, the ETEM model for the GM region has been calibrated to reproduce 2015 statistics. We have also ensured that the model reproduces statistics available for 2020. Besides, results for GHG emissions and for primary and final energy, for the projection periods (2025–2050) in our “business as usual” scenario (see Section 4, below) have been made consistent with the ones computed by NATEM-Quebec, which is a more detailed bottom–up energy model we have developed for the province of Quebec to which the GM region belongs; see [36,37,38].

## 4. Scenarios

Quebec aims to achieve 37.5% GHG emission reduction by 2030 (compared with 1990 levels), and to reach carbon neutrality by 2050 [9]. Motivated by this target, we define four scenarios by imposing different CO_2_ emission constraints on the main energy subsectors, including secondary energy generation, transportation, commercial, and residential. More specifically, the CO_2_ constraint imposes a maximum emission ceiling to the main generation units and final energy consumption sectors. The generation units include power plants, biofuel, and oil product generation units. The energy consumption sectors include transportation (light-duty vehicles, public transportation, trains, and metros), commercial, and residential (heating and cooling demand). Below, we give the details of each scenario:

- Business as usual (BAU): This scenario is a reference scenario that includes all current provincial policies, such as governmental financial incentives for a large adoption of electric vehicles. However, this scenario imposes no limitation on GHG emissions. In other words, this scenario is a disengagement from the state targets in the sense that no further climate measures are enforced beyond those already in place.
- GHG1: A GHG emission reduction scenario with a 37.5% reduction target by 2030, and a 53% reduction target by 2050 (relative to 1990).
- GHG2: A more stringent reduction scenario with a 37.5% GHG-reduction target by 2030, and continuing the same reduction trend until 2050, which yields a 73% emission reduction (relative to 1990).
- GHG3: A deep decarbonization scenario which assumes a linear GHG reduction to achieve a 44% reduction by 2030 and a 93% reduction by 2050 (relative to 1990).

## 5. Results

In this section, we present the main results obtained. Our purpose is to show how each sector contributes to the GHG emission reduction, what the main technological changes are, and how the pattern of primary energy consumption changes.

### 5.1. GHG Emissions

**Figure 3:**
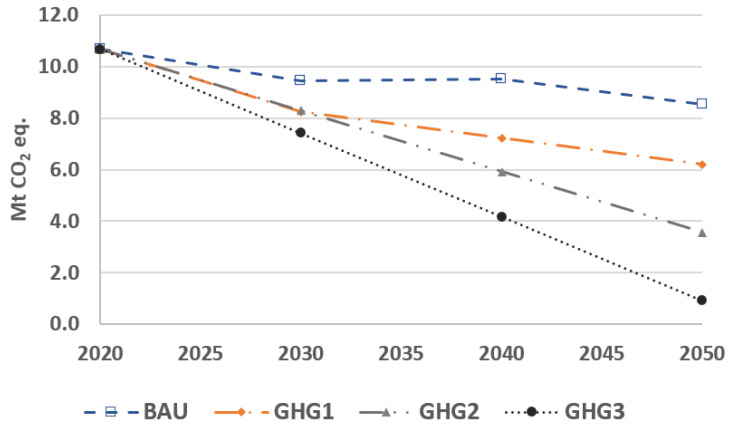
Total GHG emissions for different environmental scenarios.

In this section, we present the total GHG emissions of the transportation, residential, and commercial sectors, and evaluate how each sector contributes to the reduction target. Figure 3 shows the total emission of these sectors from 2020 to 2050. In the GHG1, GHG2, and GHG3 scenarios, emission reductions follow the emission constraints. Besides, emissions are decreasing in the BAU scenario due to the increasing share of electric-based vehicles in the transportation sector, but partly offset by the growing fossil fuel consumption in the residential and commercial sectors.

**Figure 4:**
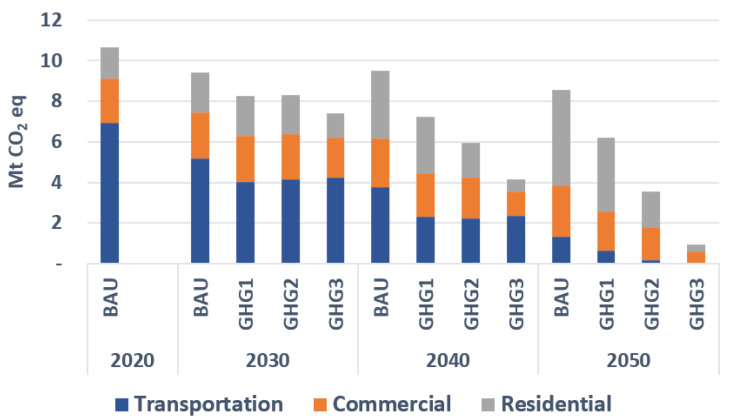
Breakdown of GHG emission reductions by sectors and environmental scenarios.

Figure 4 gives next the breakdown of emissions by sector. It shows that transportation with 6.9 Mt CO_2_-eq has the largest share in 2020 (60% of the total), as the current transportation system mostly relies on petroleum fuels. Over time, the share of the electric vehicles (EVs) increases following an assumed price reduction for these technologies. This price reduction is partly due to governmental incentives to promote the purchase EVs, and partly to a long-term technological price reduction. Consequently, the share of the transportation sector in the total GHG emission, in the BAU scenario, reduces to 15% by 2050. Imposing CO_2_ emission constraints further reduces the share of the transportation sector in the total emission by replacing conventional and hybrid cars with plug-in electric vehicles. In particular, emissions of the transportation sector reduce to almost zero in 2050 in GHG3.

Emissions of the residential sector increase in the BAU scenario as the ETEM relies mostly on natural gas in order to satisfy the heating and cooling demands. However, imposing environmental constraints forces the ETEM to electrify this sector. In particular, emissions of the residential sector reduces from 0.7 in 2020 to 0.3 Mt CO_2_-eq in 2050 in GHG3. Finally, the commercial sector is responsible for 23% of the total CO_2_ emission in 2020. In the BAU scenario, emissions of this sector increases from 2.2 in 2020 to 2.5 Mt CO_2_-eq in 2050. However, under a deep decarbonization scenario (GHG3), this sector will only emit 0.6 Mt CO_2_-eq in 2050.

**Figure 5:**
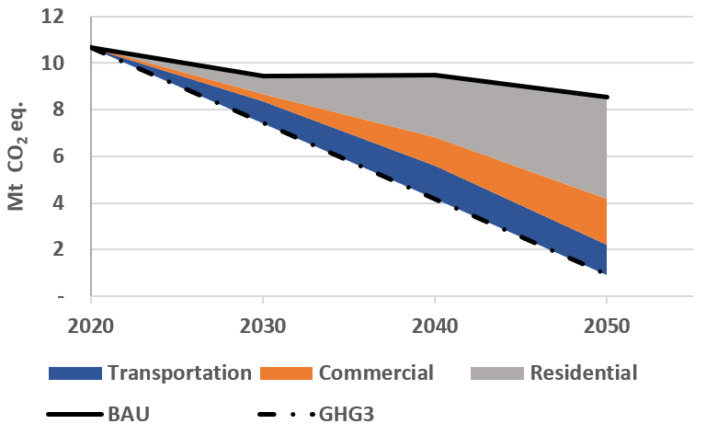
Contribution of each sector in the total GHG reductions in the deep decarbonization environmental scenario (GHG3) compared with the reference scenario (BAU).

Figure 5 depicts how each sector contributes to the total CO_2_ emission reduction when a deep decarbonization constraint (in GHG3) is imposed. Specifically, this constraint reduces the total emission to around 3 and 10 Mt CO_2_-eq in 2030 and 2050, respectively, compared with the BAU scenario. This figure also reveals that imposing such environmental constraint mostly affects the residential sector. On the other hand, the lower effect is on the transportation sector, as it is already largely decarbonized in the BAU scenario, following an assumed price reduction for EVs.

### 5.2. Final Energy Consumption

**Figure 6:**
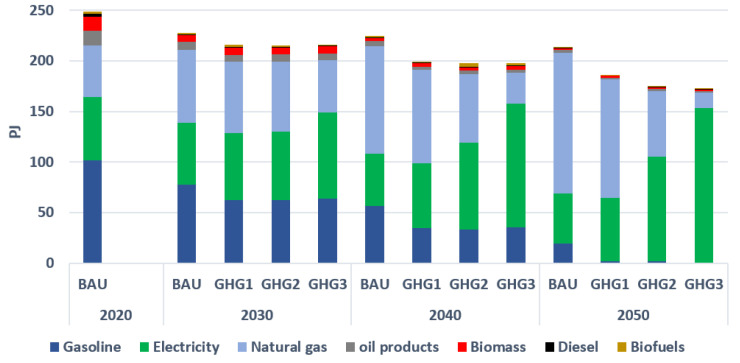
Total final energy consumption by type of energy and environmental scenarios.

Figure 6 illustrates the final energy consumption by type of fuel in the GM region under different environmental scenarios, while natural gas will be the dominant source of energy in 2050 in the BAU scenario, GHG3 proposes a combination of technologies that mostly consume electricity. Namely, electricity consumption will be tripled in 2050 compared with 2020 in GHG3. In addition, the consumption of gasoline, as one of the most important fuels in the current energy system (2020), will be gradually reduced even in BAU. More specifically, the consumption of gasoline reduces from 101 PJ in 2020 to 19.2 in 2050 in BAU, and to almost zero in GHG1, GHG2, and GHG3. Besides the shift in the primary energy composition, the total level of consumption is also affected by the environmental constraint scenarios. For example, total primary energy used in GHG3 is 13% and 30% less than in BAU in 2030 and 2050, respectively. The reason is that imposing environmental constraints encourages to invest on more efficient technologies. In addition, total primary energy used in the residential and commercial sectors decreases because of investments in buildings insulation.

**Figure 7:**
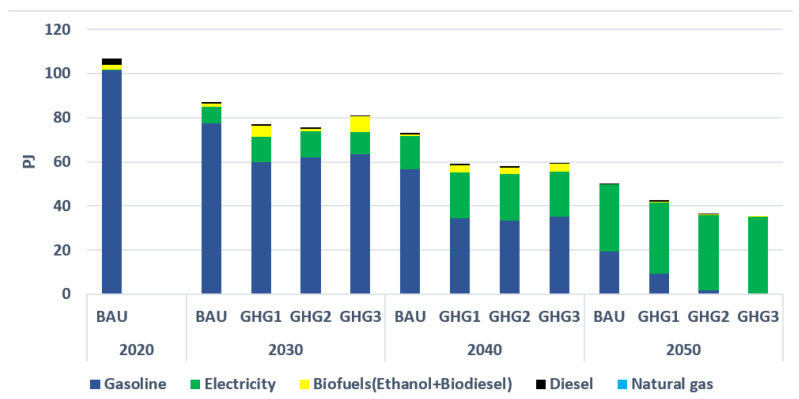
Final energy consumption by type in the transport sector.

With around 108 PJ in 2020, the transportation sector is the largest primary energy consumer in the GM region (compared with the residential and commercial sectors). In addition, gasoline, with 101 PJ, is the dominant fuel in this sector in 2020 (see Figure 7). Diesel and ethanol are the second and third fuels with 3 PJ and 2.1 PJ, respectively, in 2020. Finally, light-duty vehicles consume the largest amount of energy compared with other transportation vehicles, such as public buses and metros. Figure 7 also indicates that the consumption of gasoline and diesel is gradually reduced over time, as these fuels are substituted by electricity, even in BAU. This is due to (i) an assumed increasing cost of fossil-based fuels; and (ii) an assumed price reduction in electric hybrid vehicles over time, as a consequence of governmental incentives to promote these cars and the long-term lowering in the price of hybrid and electric cars. In addition, stringent environmental constraints (in GHG1, GHG2, and GHG3, respectively) result in bigger electrification rates for the transportation sector. Finally, a transition to electricity reduces total energy consumption in this sector due to the higher efficiency of EVs and electric public buses.

**Figure 8:**
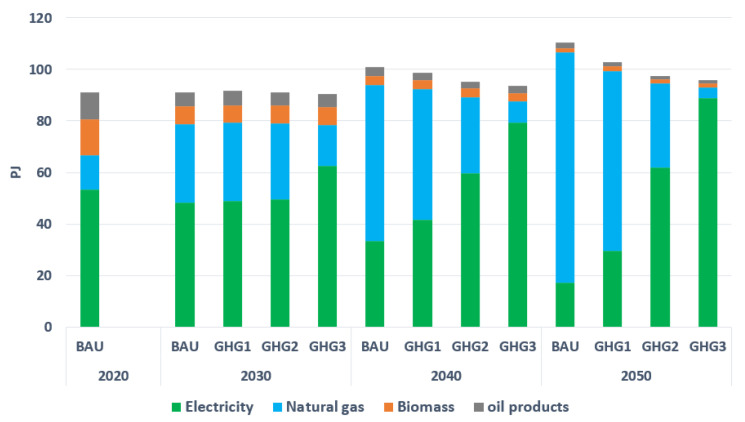
Final energy consumption by type in the residential sector.

The residential sector is the second largest energy consumer in the GM region in 2020, with a primary energy consumption of around 91 PJ to satisfy heating and cooling demands. Electricity corresponds to around 58% of total energy consumption in 2020, followed by biomass, natural gas, and oil products with 15.4%, 14.8%, and 11.5%, respectively, (see Figure 8). However, by 2050, in BAU, natural gas dominates. More precisely, natural-gas-based furnaces and stoves are the main technologies to satisfy heating demands, followed by electrical baseboard heaters. This is due to a lower marginal cost of natural gas compared with electricity. However, imposing more restrictions on CO_2_ emissions (in GHG1, GHG2, and GHG3), encourages a higher penetration of electrical baseboard heaters and heat pumps, and consequently reduces further the share of natural gas in total energy consumption. This also triggers larger investments in residential buildings insulation, which reduces final energy consumption. Finally, one can note that the electrification rate is highly sensitive to the severity of the CO_2_ reductions, with by 2050 levels of 28%, 63%, and 92%, in GHG1, GHG2, and GHG3, respectively. Transitioning to near-zero emissions in this sector requires thus stringent environmental restrictions.

**Figure 9:**
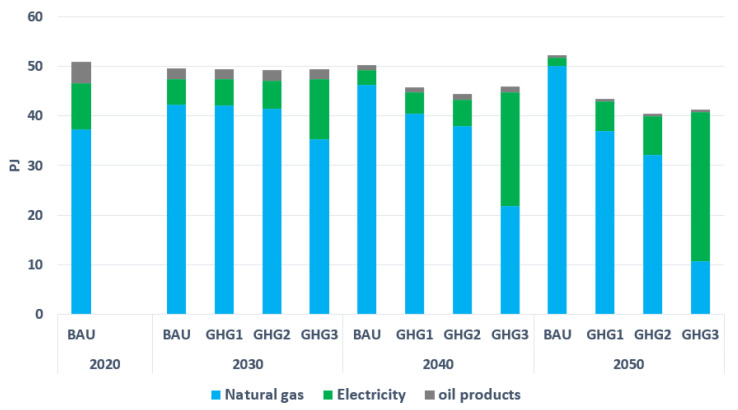
Final energy consumption by type in the commercial sector.

Concerning the commercial sector, it uses 50.8 PJ of primary energies in 2020. Natural gas, with 73% of total energy consumption, is dominant in 2020. Electricity and oil products are second and third with 18% and 9%, respectively, (see Figure 9). With no or less-stringent environmental restrictions (in BAU, GHG1, and GHG2), natural gas remains dominant by 2050. Similarly to the residential sector, this is due to a lower marginal cost for natural gas compared with electricity. However, under a deep decarbonization (GHG3), a transition from fossil-based furnaces to electrical heat pumps takes place to satisfy heating demands. The share of electricity in this scenario thus increases from 18% in 2020 to 71% in 2050. In addition, imposing environmental constraints (in GHG1, GHG2, and GHG3) increases investments in commercial buildings insulation, and thus reduces final energy consumption compared with BAU.

### 5.3. Sensitivity Analysis

As seen in Section 5.1, the transportation sector plays an important role when decarbonizing the energy sector. On the other hand, total energy consumption in the transportation sector, and accordingly total CO_2_ emissions, depend on the mode of transportation. If a larger share of the mobility demand is met by public transportation facilities, the required trip per passenger-kilometer demand reduces, resulting in lower primary energy consumption. In this section, we evaluate the sensitivity of our results to the share of each transportation mode (public and private) in the total mobility demand. To do so, we exogenously shift 20% and 50% of the mobility demand from light-duty vehicles to public transport (buses and metros). These transport modal shifts start in 2030 and continue until 2050.

**Figure 10:**
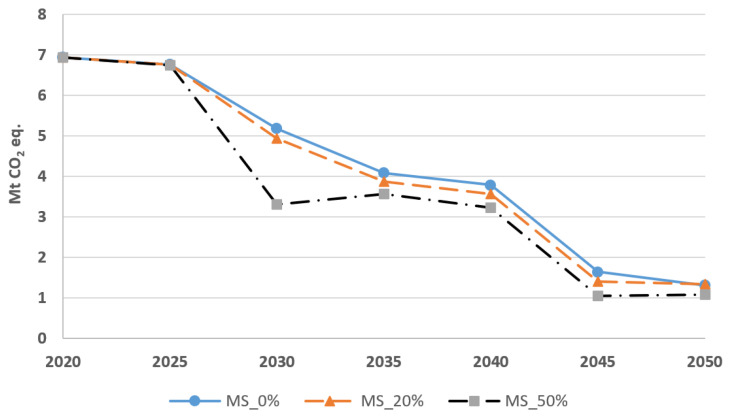
GHG emissions from the transportation sector for different modal shifts in BAU, as the mobility demand is partially shifted from light-duty vehicles to public transportation.

Figure 10 compares total CO_2_-eq emissions of the transportation sector under the different modal shifts, while MS_0% represents the default modal share, and MS_20% and MS_50% correspond to the 20% and 50% modal shifts, respectively. This figure relates to the BAU scenario. In 2030, total emissions under MS_50% are 36% lower than in MS_0%. However, this emission gap reduces afterwards following the electrification of light-duty vehicles. In other words, shifting to public transportation can be a temporary strategy to reduce emissions before electrifying the transportation sector.

**Figure 11:**
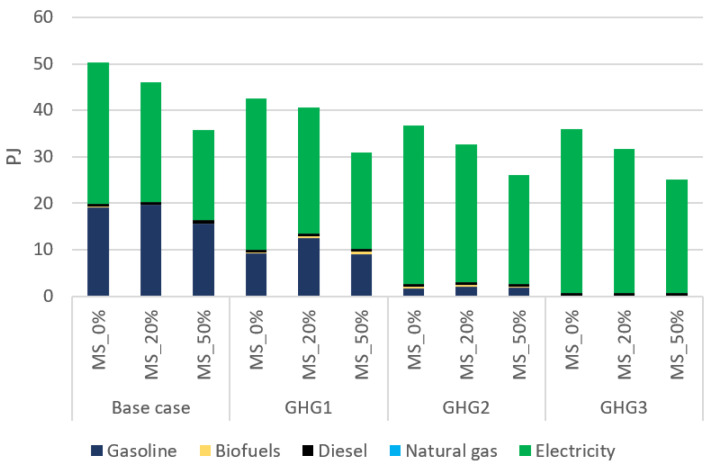
Final energy consumption for the transportation sector in 2050 under different scenarios and transport modal shifts.

Figure 11 compares next final energy consumption of the transportation sector, in 2050, under different scenarios and modal shifts. In general, a shift from private to public transportation not only reduces primary energy consumption, but also increases the share of electricity in final energy consumption. The former trend is because buses and metros are able to move more passengers with a lower energy consumption per passenger. The latter is due to the higher electrification rate in public transportation compared with the private one. Namely, a large portion of public transportation is already electrified thanks to metros. In addition, because the number of buses used is much lower than the one of private cars, electrifying them is faster and easier. Therefore, shifting to public transportation reduces fossil fuels consumption.

## 6. Discussion

In this paper, we examine different decarbonization pathways for the GM region, and evaluate their impacts on the energy sector. Results show that the GM region can reach a near zero-emission energy sector by electrifying the transportation, commercial and residential sectors, and by increasing the energy efficiency of buildings. This is mainly because (i) the majority of electricity produced in Quebec is carbon-free coming from low-cost hydropower, and (ii) because there are limited sources of industrial emissions in the region. In addition, when shifting from private to public transportation, final energy consumption can be further reduced.

With 30% of the total energy-related emissions in Quebec, road transportation is currently the largest source of GHG emissions in the GM area [9,10]. In addition, gasoline is the main primary energy used in this sector with a 94% share. Our findings suggest that promoting the use of EVs reduces the share of gasoline. In particular, under a deep decarbonization scenario (GHG3), this share drops to 78% by 2030, 59% by 2040, and 0% by 2050. In addition, a (partial) shift from private to public transportation can reduce total energy consumption of the transportation sector by up to 30%. Our modal shift scenarios are in line with a city goal to shift around 25% of private transport to public transport. Residential and commercial buildings account for 28% of GHG emissions in the GM area [9]. Our results suggest that lowering emissions can be achieved through electric heating and cooling technologies, in particular baseboard heaters and heat pumps. In parallel, investments in energy efficiency lead to a decrease in total energy consumption. Here again, these findings are in line with the GM plan [9] in terms of energy efficiency improvements for residential and commercial buildings.

Finally, our results are consistent with the main strategies proposed in other related papers. For instance, according to [26], Mexico city plans to reach ambitious GHG emission reductions by using more renewable energies, energy efficiency improvements, and shifting toward cleaner and electrified public transport. Likewise, as presented in [27], the city of Oslo has ambitious decarbonization targets (i.e., 50% GHG emission reduction by 2030, and no fossil fuels by 2050), and it can meet the target by (i) shifting from private to public transportation powered by renewable energy, (ii) electrification of heating systems, and (iii) energy efficiency improvements.

We acknowledge some important limitations of this paper, as follows: (i) we do not explicitly consider uncertainties in the energy system (uncertainty of prices, demands, technological innovations, etc.); (ii) we do not carry out a life cycle GHG emission assessment, and as a result, our model does not consider the emissions resulting from constructing facilities (such as CO_2_ emitted from increasing utilization of concrete); (iii) we abstract some details of the energy system, such as technological choices in industry, agriculture, and specific consumption in the transportation, residential, and commercial sectors; and (iv) we have not considered some modes of transportation, such as bikes and e-bikes.

## 7. Conclusions

In this paper, we adopted the formulation of the ETEM (Energy–Technology–Environment Model) to assess the long-term energy transition of the Greater Montreal (GM) region in Quebec. The proposed model covers the years 2020–2050 and provides insights on the capacity expansion of generation technologies, demand-side technological shifts, total primary energy consumption, and total energy-related CO_2_-eq emissions. We evaluated the impact of imposing different CO_2_-eq emission reduction constraints on energy transition pathways for the GM region. Results show that the transportation sector, with a 6.9 million ton (Mt) emission reduction compared with the 2020 level, plays an important role in a deep decarbonization (GHG3 scenario) of the GM region. This reduction can be achieved by electrifying private and public vehicles. Moreover, commercial and residential sectors will contribute to the deep decarbonization by, respectively, reducing 1.6 Mt and 1 Mt CO_2_-eq (compared with their 2020 levels). The most important decarbonization strategies in these sectors include (i) replacing fossil-fuel-based furnaces with electric-based heat pumps to satisfy heating demands, and (ii) reducing energy consumption by increasing buildings insulation.

Several directions could be considered for future work. First, it is worth explicitly considering the uncertainty in our analysis. Because of the long-term horizon of planning in the ETEM, there are many sources of uncertainty that affect results. Demands, prices of fuels and technologies, and efficiency of technologies are among these uncertain influential parameters. Therefore, a first direction is to consider these uncertainties and obtain results that are robust against perturbations of these parameters. Second, our analysis does not model a complete list of hydrogen technologies. Given the potential importance of hydrogen-based technologies in different sectors, it is worthwhile investigating the energy transition considering as well all these technologies. Finally, expanding the boundaries of the energy system to include a detailed description of industrial and agricultural technology choices could be a subject for future research.

## Data Availability

Both the ETEM model and the GM region database can be made accessible upon reasonable request to the authors.

## Nomenclatures

The following nomenclatures are used in this manuscript:

t∈T	Index for time period	$\beta_{t,s,p}$	Capacity factor
s∈S	Index for time slices	$\eta_{c}$	Network efficiency
p∈P	Index for technologies	$\eta_{f,f^{\prime }}^{t}$	Technology efficiency
c∈C	Index for energy commodities	$\lambda_{t,s,l,l^{\prime },c}^{\prime \prime }$	Transmission cost
c∈CS	Index for energy storage	$\lambda_{t,s,c}^{\prime }$	Export cost
f∈F	Index for energy flows	$\lambda_{t,s,c}$	Import cost
l∈L	Index for buses (geographical zones)	$\nu_{t,s,c}$	Maximum deviation from
j∈J	Index for seasons		nominal demand response
i∈I	Index for period-seasons (t,j)	$\nu_{t,p}$	Variable cost
$P_{c}^{C}\subseteq P$	Set of technologies consuming *c*	$\Omega_{t,l,p}$	Available capacity of technology *p*
$P_{c}^{P}\subseteq P$	Set of technologies producing *c*	$\pi_{t,p}$	Fixed production cost
$P^{R}\subseteq P$	Set of intermittent technologies	ρ	Discount factor
$C^{I}\subseteq C$	Set of imported commodities	$\theta_{p}^{c}$	Proportion of output *c* from technology
$C^{D}\subseteq C$	Set of useful demands		*p* that can be used in peak period
$C^{EX}\subseteq C$	Set of exported commodities	$l_{p}$	Life duration of technology *p*
$C^{TR}\subseteq C$	Set of transmitted commodities	$\Theta_{t,l,d}$	Annual final demand
$C_{f}\subseteq C$	Set of commodities linked to flow *f*	$\upsilon_{t,s,c}$	Nominal demand response
$C^{G}\subseteq C$	Set of commodities with margin reserve	$\varrho_{t,s,c}$	Required reserve for commodity $c\in C^{G}$
$S^{j}\subseteq S$	Set of time slices *s* in season *j*	$C_{t,l,p}$	Variable for new capacity addition
$S^{s}\subseteq S$	Set of successive time slices of *s*	$C_{t,l,p}^{T}$	Total installed capacity
$S^{G}\subseteq S$	Set of time slices in peak period	$P_{t,s,l,p,c}$	Variable for activity of technology *p*
${\mathrm{FI}}_{p}\subseteq F$	Set of inputs to technology *p*	$I_{t,s,l,c}$	Variable for import
${\mathrm{FO}}_{p}\subseteq F$	Set of outputs from technology *p*	$E_{t,s,l,c}$	Variable for export
$\alpha_{t,p}$	Investment cost	$T_{t,s,l,l^{\prime },c}$	Variable for regional transmission
		$V_{t,s,l,d}$	Variable for demand response

## Appendix A. ETEM Formulation

In this section, we briefly present the main equations of the ETEM to highlight its logic. Note again that a full description of the ETEM can be found in [11,31]. Here, we mostly followed the notation used in [32]. Namely, index t∈T indicates time periods, and s∈S is the set of time slices inside a period. Index c∈C refers to energy commodities, and p∈P is reserved for technologies. Input–output energy flows are shown by index f∈F, and finally, set L identifies different geographical zones. In the ETEM, there are 5 main variables. $P_{t,s,l,p,c}$ is the level of energy commodity, *c*, which is produced or consumed, in period, *t*, time slice, *s*, by technology, *p*, which is installed in region *l*. $C_{t,l,c}$ is the newly installed capacity of technology *p* in region *l*. $I_{t,s,l,c}$ and $E_{t,s,l,c}$ are the level of import and export of commodity *c* in region *l*, period *t* and time slice *s*. $T_{t,s,l,l^{\prime },c}$ is the transmission of energy *c* between regions *l* and $l^{\prime }$. Finally, $V_{t,s,d}$ is a demand response variable which optimally distribute annual demand between different time slices. Nomenclatures summarizes and provides a definition for all indices, parameters, and variables used in this appendix.

Equation (A1) presents the objective function of the ETEM. It minimizes a total discounted cost of the system which includes (i) investment cost, (ii) fixed cost, (iii) variable cost, (iv) net import cost, and finally (v) transmission cost.

(A1) $$\begin{array}{cc} W^{*}:= & \min\sum_{t,l}\rho^{t}\cdot \left(\sum_{p}\alpha_{t,p}C_{t,l,p}+\pi_{t,p}\left(\sum_{k=0}^{l_{p}-1}C_{t-k,l,p}+\Omega_{t,l,p}\right)+\sum_{s,c}\nu_{t,p}P_{t,s,l,p,c}+\right.\\ & \;\;\left.\sum_{s,c}\left(\lambda_{t,s,c}I_{t,s,l,c}-\lambda_{t,s,c}^{\prime }E_{t,s,l,c}\right)+\sum_{s,l^{\prime },c}\lambda_{t,s,l,l^{\prime },c}^{{}^{\prime \prime }}T_{t,s,l,l^{\prime },c}\right) \end{array}$$

Equation (A2) presents balance constraints. This equation guarantees that the procurement of energy, for each energy commodity, *c*, is always greater than its consumption. The procurement of energy is the summation of the internal production, import, and the net transmission of the energy (left hand side of the constraint). Similarly, the consumption of energy commodity *c* consists of internal consumption and export.

(A2) $$\begin{array}{cc} & \left(\sum_{p\in P_{c}^{P}}P_{t,s,l,p,c}+I_{t,s,l,c}\right)\eta_{c}+\sum_{l^{\prime }\neq l}\left(\eta_{c}T_{t,s,l^{\prime },l,c}-T_{t,s,l,l^{\prime },c}\right)\geq \sum_{p\in P_{c}^{C}}P_{t,s,l,p,c}+E_{t,s,l,c}\\ & \forall \;t,s,l,c\in C/C^{D} \end{array}$$

Equation (A3) models the concept of energy storage in the ETEM. Specifically, the storable energy commodity c∈CS is produced and stored in time slice *s*, and it is retrieved, by considering its efficiency factor $\eta_{c}$, in subsequent time slice $s^{\prime }$.

(A3) $$\begin{array}{cc} & \eta_{c}\sum_{p\in P^{P_{c}}}P_{t,s,l,p,c}=\sum_{p\in P^{C_{c}},s^{\prime }\in S^{s}}P_{t,s^{\prime },l,p,c}\;\forall t,s,l,c\in \mathrm{CS} \end{array}$$

Equations (A4)–(A6) model the demand and demand response in the ETEM. Equation (A4) simply imposes the total procurement of energy service $c\in C^{D}$ to be greater than its total demand. In this equation, the variable $V_{t,s,c}$ optimally distributes the total demand into different time slices. Equation (A5) imposes the demand response variable to be inside a box around the nominal values for the demand response. Finally, Equation (A6) imposes the summation of the demand response for a specific season to be equal to the summation of the nominal values for the demand response. Indeed, this equation guarantees that the shift in the demand consumption from one time slice to another time slice does not transfer from one season to another season.

(A4) $$\begin{array}{cc} & \sum_{p\in P_{c}^{P}}P_{t,s,l,p,c}\geq \Theta_{t,l,c}V_{t,s,c}\;\forall t,s,l,c\in C^{D} \end{array}$$

(A5) $$\begin{array}{cc} & \upsilon_{t,s,c}\left(1-\nu_{t,s,c}\right)\leq V_{t,s,c}\leq \upsilon_{t,s,c}\left(1+\nu_{t,s,c}\right)\;\forall t,s,c\in C^{D} \end{array}$$

(A6) $$\begin{array}{cc} & \sum_{s\in S_{j}}V_{t,s,c}=\sum_{s\in S_{j}}\upsilon_{t,s,c}\;\forall t,c\in C^{D},j\in J \end{array}$$

Equation (A7) models the capacity factor of each technology. In this equation, the production of each technology is limited by the product of a capacity factor with the total available capacity of that technology.

(A7) $$\begin{array}{cc} & \sum_{c:p\in P_{c}^{P}}P_{t,s,l,p,c}\leq \beta_{t,s,p}\left(\sum_{k=0}^{l_{p}-1}C_{t-k,l,p}+\Omega_{t,l,p}\right)\;\forall t,s,l,p\notin P^{R}, \end{array}$$

Finally, Equation (A8) models the efficiency of each technology. Specifically, in this equation, the output of each technology is equal to a portion of the total energy input to that technology.

(A8) $$\begin{array}{cc} & \sum_{c\in C_{f^{\prime }}}P_{t,s,l,p,c}=\eta_{f,f^{\prime }}^{t}\cdot \sum_{c\in C_{f}}P_{t,s,l,p,c}\;\forall f\in {\mathrm{FI}}_{p},f^{\prime }\in {\mathrm{FO}}_{p},t,s,l,p \end{array}$$

## Appendix B. GM Energy Database

A database for the energy system of the Greater Montreal (GM) region has mostly been extracted, aggregated, and calibrated from the following sources: (i) database of the North American TIMES Energy Model (NATEM) [35]; (ii) statistics Canada [39,40]; (iii) the Comprehensive Energy Use Database produced by the Canadian Office of Energy Efficiency [41]; (iv) Canadian energy outlooks produced by the Canada Energy Regulator (CER) [42]; and (v) national reports produced by Environment Canada [43].

The GM energy database includes information on 130 technologies (including 8 conversion technologies, and 90 end-use technologies). Moreover, 51 energy commodities are explicitly modeled. In particular, the database considers 13 types of final energy demand, and the possibility to import 18 primary and secondary energies (including different oil derivatives, electricity…).

The ETEM considers wind power, hydro power and solar power as the renewable resources, and crude oil, coal, and natural gas as fossil fuel resources. Besides the import of crude oil and diesel, the ETEM also considers the import of oil-derived fuels, including gasoline, diesel, light fuel oil, heavy fuel oil, petroleum coke, jet fuel. Biomass, biogas, biodiesel and bioethanol could also be produced inside the system.

Final energy demand is categorized into residential, commercial, transportation, agricultural, and industrial. Residential and commercial demands are divided into demands for heating, cooling, and other types of demand (including appliances, lighting, etc.). To satisfy commercial and residential heating, different possible technologies are considered, including natural gas furnaces, electrical plinth and furnaces, electrical heat pump, and fossil fuel heat pumps. For residential and commercial cooling, two types of central and individual coolers are considered, based on natural gas or electricity. Transportation demand is divided into light-duty vehicles, metro, public transportation, train, and other types of transportation (including maritime transportation, and air transportation). In the transportation sector, different vehicles are considered, including fossil fuel cars and buses, as well as electric and hybrid buses and cars. Table A1 summarizes the demand classification and the number of technologies in each class of demand.

The resulting ETEM model for the GM region covers a period extending from 2015 to 2050, with 8 periods of 5 years. It also considers 95 intraperiod time slices (4 s and 24 h for each day in each season) to capture the pattern of production and consumption of electricity. This database is available as an AMPL data file [44]. Compiling the ETEM with the GM region database results in a linear optimization problem with 1,069,274 constraints, and 1,300,714 variables, which is solved using the IBM CPLEX optimizer [45].

**Table A1:** Useful demand classification.

Sector	Demand Type	Unit	Number of Technologies	Fuels ^1^
residential	Heating	TJ	17	NGA, ELC, LFO, PRO, BIO
	Cooling	TJ	6	ELC
	Other	TJ	2	NGA, ELC, LFO, PRO, BIO
Commercial	Heating	TJ	21	NGA, ELC, HET, LFO, HFO, PRO
	Cooling	TJ	9	NGA, ELC
	Other	TJ	10	NGA, ELC, HET, LFO, HFO, PRO
Trnasportation	Light-duty vehicles	tkmv/d	7	GSL, ETH, ELC, DST, BSL
	Public transportation	tkmv/d	10	GSL, ETH, ELC, DST, BSL, NGA
	Metro	tkmv/d	2	ELC
	Train	tkmv/d	2	DST, BSL
	Other	tkmv/d	1	GSL, ETH, ELC, DST, BSL, NGA, LFO, HFO, ATR

^1^ ATR—jet fuel; BIO—biomass; BSL—biodiesel; DST—diesel; ELC—electricity; ETH—ethanol; GSL—gasoline; HET—centrally produced heat; HFO—heavy fuel oil; LFO—light fuel oil; PRO—propane; NGA—natural gas.
